# Mitochondrial DNA mutations in Malaysian female breast cancer patients

**DOI:** 10.1371/journal.pone.0233461

**Published:** 2020-05-22

**Authors:** Raevathi Omasanggar, Choo Yee Yu, Geik Yong Ang, Nor Aina Emran, Normayah Kitan, Anita Baghawi, Atiki Falparado Ahmad, Maizaton Atmadini Abdullah, Lay Kek Teh, Sandra Maniam

**Affiliations:** 1 Department of Human Anatomy, Faculty of Medicine and Health Sciences, Universiti Putra Malaysia, Serdang, Selangor, Malaysia; 2 Integrative Pharmacogenomics Institute (iPROMISE), Universiti Teknologi MARA, Bandar Puncak Alam, Puncak Alam, Selangor, Malaysia; 3 Faculty of Sports Science and Recreation, Universiti Teknologi MARA, Shah Alam, Selangor, Malaysia; 4 Department of General Surgery, Hospital Kuala Lumpur, Kuala Lumpur, Malaysia; 5 Department of General Surgery, Hospital Putrajaya, Putrajaya, Malaysia; 6 Department of General Surgery, National Cancer Institute, Putrajaya, Malaysia; 7 Department of Pathology, Faculty of Medicine and Health Sciences, Universiti Putra Malaysia, Serdang, Selangor, Malaysia; 8 Laboratory of Molecular Medicine, Institute of Bioscience, University Putra Malaysia, Selangor, Malaysia; German Cancer Research Center (DKFZ), GERMANY

## Abstract

Cancer development has been ascribed with diverse genetic variations which are identified in both mitochondrial and nuclear genomes. Mitochondrial DNA (mtDNA) alterations have been detected in several tumours which include lung, colorectal, renal, pancreatic and breast cancer. Several studies have explored the breast tumour-specific mtDNA alteration mainly in Western population. This study aims to identify mtDNA alterations of 20 breast cancer patients in Malaysia by next generation sequencing analysis. Twenty matched tumours with corresponding normal breast tissues were obtained from female breast cancer patients who underwent mastectomy. Total DNA was extracted from all samples and the entire mtDNA (16.6kb) was amplified using long range PCR amplification. The amplified PCR products were sequenced using mtDNA next-generation sequencing (NGS) on an Illumina Miseq platform. Sequencing involves the entire mtDNA (16.6kb) from all pairs of samples with high-coverage (~ 9,544 reads per base). MtDNA variants were called and annotated using mtDNA-Server, a web server. A total of 18 of 20 patients had at least one somatic mtDNA mutation in their tumour samples. Overall, 65 somatic mutations were identified, with 30 novel mutations. The majority (59%) of the somatic mutations were in the coding region, whereas only 11% of the mutations occurred in the D-loop. Notably, somatic mutations in protein-coding regions were non-synonymous (49%) in which 15.4% of them are potentially deleterious. A total of 753 germline mutations were identified and four of which were novel mutations. Compared to somatic alterations, less than 1% of germline missense mutations are harmful. The findings of this study may enhance the current knowledge of mtDNA alterations in breast cancer. To date, the catalogue of mutations identified in this study is the first evidence of mtDNA alterations in Malaysian female breast cancer patients.

## Introduction

Breast cancer is the most common malignancy in women and one of the leading causes of cancer-related deaths in women worldwide, with nearly 2.1 million new cases estimated and responsible for the deaths of 629,679 women in 2018 [1]. Breast cancer incidences in Malaysia are estimated to be 7,593 (32.7%) new cases in 2018, with 2,894 deaths [1]. Despite various emerging treatment strategies and novel therapies in treating breast cancer, it is estimated that one in 30 Malaysian women is at risk of breast cancer in her lifetime [2]. Studies involving analysis of large samples and controls led to identification of genetic factors involved in predisposition to breast cancer [3, 4]. Albeit numerous studies reported the association of DNA mutations in cancer mainly in Western population, limited research exploring the effects of mitochondrial DNA (mtDNA) alterations specifically in Asian population were documented.

Mitochondria are the energy synthesising organelles in the cells. They are responsible for the generation of adenosine triphosphate (ATP) molecules through oxidative phosphorylation (OXPHOS). Each mitochondrion contains genetically compact circular double-stranded mtDNA loops with an average of 100–10,000 copies per cell at a high copy number. The mtDNA copy number per cell varies widely in different human tissues and is maintained to meet the energy requirement to sustain normal physiological functions of the cell [5]. mtDNA consists of 16,569 base pairs which contains 37 essential genes that encode for 13 respiratory chain subunits essential for the OXPHOS system, 22 transfer RNAs (tRNAs) and two ribosomal RNAs (rRNAs), namely 12S and 16S that are required for the transcription and translation of mitochondrial proteins [5].

It is increasingly apparent that mitochondria play a pivotal role in modulating oncogenesis by virtue of their key functions in energy production, synthesis of building blocks for tumour anabolism, generation of reactive oxygen species (ROS), regulation of cell death and immunosurveillance [6, 7]. The mtDNA plays an important role in regulating mitochondrial functions and are highly susceptible to damage. MtDNA is maternally inherited in which the integrity of all copies is maintained in the same sequence, a state known as “homoplasmy” [8]. However, these mtDNAs may undergo sequence variations and each cell may contain different proportions of mutant and normal (wild-type) mtDNAs during mitotic division, a state known as “heteroplasmy” [9]. The damaging effects of ROS radicals produced in the electron transport chain (ETC), replication cycle defects and lack of protective DNA repair mechanisms are known to contribute to the variation of the mtDNA gene sequence [10–12].

Various types of mtDNA mutations have been detected in breast cancers [13, 14]. Common genetic changes in mtDNA are germline and somatic mutations which include gene deletions, missense mutations, frame-shift mutations and insertions [15]. Both germline and somatic mutations are implicated in breast tumour formation. Germline mutations were shown as risk factor for invasive breast cancer whilst most of the mutations identified in breast cancers were somatic mutations [15, 16]. Mutations in mtDNA are known to perturb the OXPHOS system in various cancer cells [17]. Downregulation of OXPHOS activity was shown to be involved in the early stages of carcinogenesis as well as in the metastases of breast neoplastic cells [18, 19]. Interestingly, A10398G polymorphism of complex I subunit ND3 of the OXPHOS system has been associated with an increased risk of breast cancer in Malaysia [20]. Although the prevalence of this polymorphism is significant, there are very limited studies exploring the entire mtDNA genome of breast cancer patients in Malaysia.

In the current study, the entire mtDNA of adjacent normal-tumour pairs of 20 patients with breast cancer was screened using in-depth coverage provided by next generation sequencing (NGS) to catalogue the mtDNA mutations. Understanding the mtDNA alterations may add to the current knowledge of mitochondrial impairment in breast cancer. The relationship of mutations burden and clinical variables of the patients were also assessed. The outcome of this first preliminary study involving Malaysian breast cancer patients will potentially serve as a reference for future studies involving the entire mtDNA in breast cancer.

## Materials and methods

### Human breast cancer tissue samples collection

This study was approved by the Medical Research & Ethics Committee (MREC) of the National Institutes of Health Malaysia [NMRR ID-15-2085-28181 (IIR)] and Ethic Committee for Research Involving Human Subjects of Universiti Putra Malaysia (JKEUPM). Written informed consent was obtained from all participants and the study was conducted according to the ethical guidelines of the Helsinki Declaration. A total of 20 female breast cancer patients were recruited between April 2016 and December 2016 from Hospital Kuala Lumpur, National Cancer Institute and Hospital Putrajaya in Peninsular Malaysia. Breast tissue samples were obtained during surgery whilst the demographic and clinical data were extracted from the medical records. Women who were more than 18 years old, pathologically confirmed with primary breast carcinoma and underwent mastectomy surgery for breast cancer were included. Patients receiving neoadjuvant chemotherapy/radiotherapy, with serious infection or concomitant disease and absence of informed consent were excluded from this study. Tumour tissue and its matched control were obtained during mastectomy surgery. Normal breast tissue samples were located in the different quadrant and at a sufficient distance from the tumour. All samples were immediately immersed in liquid nitrogen and stored until further use. The demographic details of patients and clinical features of the samples are summarised in S1 Table.

### Total DNA extraction

Total DNA was extracted from 50 mg of malignant and corresponding normal tissue samples using the QIAamp DNA Mini kit (Qiagen, Germany) according to the manufacturer’s instructions [21]. An ultraviolet-visible spectrophotometer (Thermo Scientific Nanodrop 2000, Thermo Scientific, United States) was used to quantify and assess the purity of DNA samples. All DNA samples were stored at -20°C until further use.

### PCR amplification of mtDNA

Long-range polymerase chain reaction (LR-PCR) was performed to purify mtDNA from isolated total DNA. LR-PCR was performed in two separate reactions to amplify two large overlapping amplicons (9.1kb and 11.2kb) using two sets of primer pairs MTL-F1 (5’ AAA GCA CAT ACC AAG GCC AC 3’); R1 (5’ TTG GCT CTC CTT GCA AAG TT 3’) and MTL-F2 (5’ TAT CCG CCA TCC CAT ACA TT 3’); R2 (5’ AAT GTT GAG CCG TAG ATG CC 3’) as previously described [22]. TaKaRa LA *Taq* kit (Takara Clontech, Mountain View CA, USA) was used for amplification. The 50 μL of PCR reaction conditions were: 1 x LA PCR buffer, 0.4 mM dNTP mixture, 0.4 μM primer pairs, 2.5 U TaKaRa LA *Taq*, and 2 ng of genomic template DNA. Thermal cycling parameters were: initial denaturation at 95°C for 3 minutes, then 30 cycles at 95°C for 30 s, 68°C for 10 s, 60°C for 15 s and 68°C for 11 minutes, followed by a final extension at 72°C for 10 minutes. Agarose gel electrophoresis was performed to validate the size of amplified PCR products. PCR products were quantified using the Qubit dsDNA HS Assay Kit (Life Technologies Corporation, Oregon USA) with the Quantus Fluorometer (Promega, USA).

### Library building and paired-end sequencing on Miseq

Indexed paired-end mtDNA libraries were prepared with the Nextera XT DNA Sample Prep Kit and the Nextera XT Index Kit (Illumina, USA) according to the manufacturer’s guidelines [23]. An input of 1ng/μl mtDNA amplicons from each of the two amplicon sets was pooled and diluted further for tagmentation on thermal cycler. Tagmentation involves fragmenting the mtDNA amplicons and tagging with adapter sequences. This is followed by amplification of tagmented mtDNA using a limited-cycle PCR program as described by the manufacturer [23]. The amplified library was purified using AMPure XP beads (Beckman Coulter, USA) to remove short library fragments. The purified library was quantified using Qubit dsDNA HS Assay Kit (Life Technologies Corporation, Oregon USA) and ran on Agilent Technology 2100 Bioanalyzer using a High Sensitivity DNA chip (Agilent, USA) to check for size distribution. An undiluted library mtDNA was normalized to the average library size (600–750 bp) for every 660 g/mol of mtDNA to produce 4 nM library mtDNA. This is followed by pooling of an equal volume of libraries of all tumour-normal sample pairs of 20 patients. Pooled libraries were denatured and diluted to 10 pM sequencing input with Miseq Reagent Kit v2 (Illumina, USA) as described by the manufacturer [24]. Illumina PhiX Control, derived from phiX174 (RF1) bacteriophage of 5386 bp circular genome was used as an Illumina sequencing positive control [25]. The sequencing reactions were performed on the Miseq V2 (2 x 250 bp) platform (Illumina, USA) in compliance with the manufacturer’s preparation guides for paired end runs [26].

### Variant calling and annotation

The Illumina Miseq paired-end FASTQ files of all 20 tumour and matched normal samples were analysed with mtDNA-Server Version 1.0.6. [27]. Server default parameters were used to generate sequence data. FASTQ input sample files were aligned to rCRS and pair reads mapped before BAM files were generated. Several filters were applied to detect homoplasmic and heteroplasmic variants from the resulting BAM files. Only reads with a mapping quality score >20, alignment quality >30 and base quality >20 were used for variants detection and 1% minor component threshold for heteroplasmies. Indels were manually classified using similar filters with the raw data file. All samples were also checked for contamination by the server.

Variants detected in both tumour and corresponding normal tissue samples of a patient were classified as germline mutations as described in previous studies [28, 29]. Variant differences between tumour and matched normal mtDNA were classified as somatic mutations [28, 29]. For somatic mutations, only variants with allele frequency <1% in the normal and ≥1% in the matched tumour samples were analysed. Germline heteroplasmies were called for alleles present in normal tissue at level ≥1%. Variants were further searched in MITOMAP [30] and dbSNP [31] to identify novel variants and to determine the clinical significance of the reported variants.

The predicted functional effects of variants were determined using precomputed values of the Polyphen2, SIFT, CADD and APOGEE algorithms, collected within the MitImpact 3D database [32] and MutPred [33] within mtDNA-Server.

### Statistical analysis and data visualization

The shift in heteroplasmy levels of heteroplasmic point mutations was analysed using a paired t-test. The distribution of germline mutations (all homoplasmic, heteroplasmic and indel mutations) and somatic mutations were assessed by dividing the mitochondrial genome into 18 regions (D-loop, other non-coding region, tRNA (all tRNA-coding genes), 12s rRNA, 16s rRNA, ATP8, ATP6, COX I, COX II, COX III, CYB, ND1, ND2, ND3, ND4,ND4L, ND5 and ND6). The proportions of mtDNA genes were calculated by dividing the total number of base pairs per gene by the number of base pairs of the entire mtDNA. The proportions of germline mutations and somatic mutations were calculated by dividing the number of mutations in a certain gene region with a total number of mutations. Testing differences in the distribution of mutations across the mtDNA genome between germline and somatic mutations were assessed using a two-sided Fisher’s exact test with rows indicating germline and somatic categories, and columns indicating regions of mutations. The association between the numbers of mutations in relation to gene size were tested for independency with a Pearson correlation analysis. Statistical test was performed using IBM SPSS version 23. A value of P<0.05 was considered significant, whereas values of P<0.01 and P<0.001 were considered highly significant. Tables were generated using Microsoft Excel 2010. Figures were generated using Microsoft Excel 2010 and Adobe Illustrator CS3.

### Study flowchart

**Fig 1:**
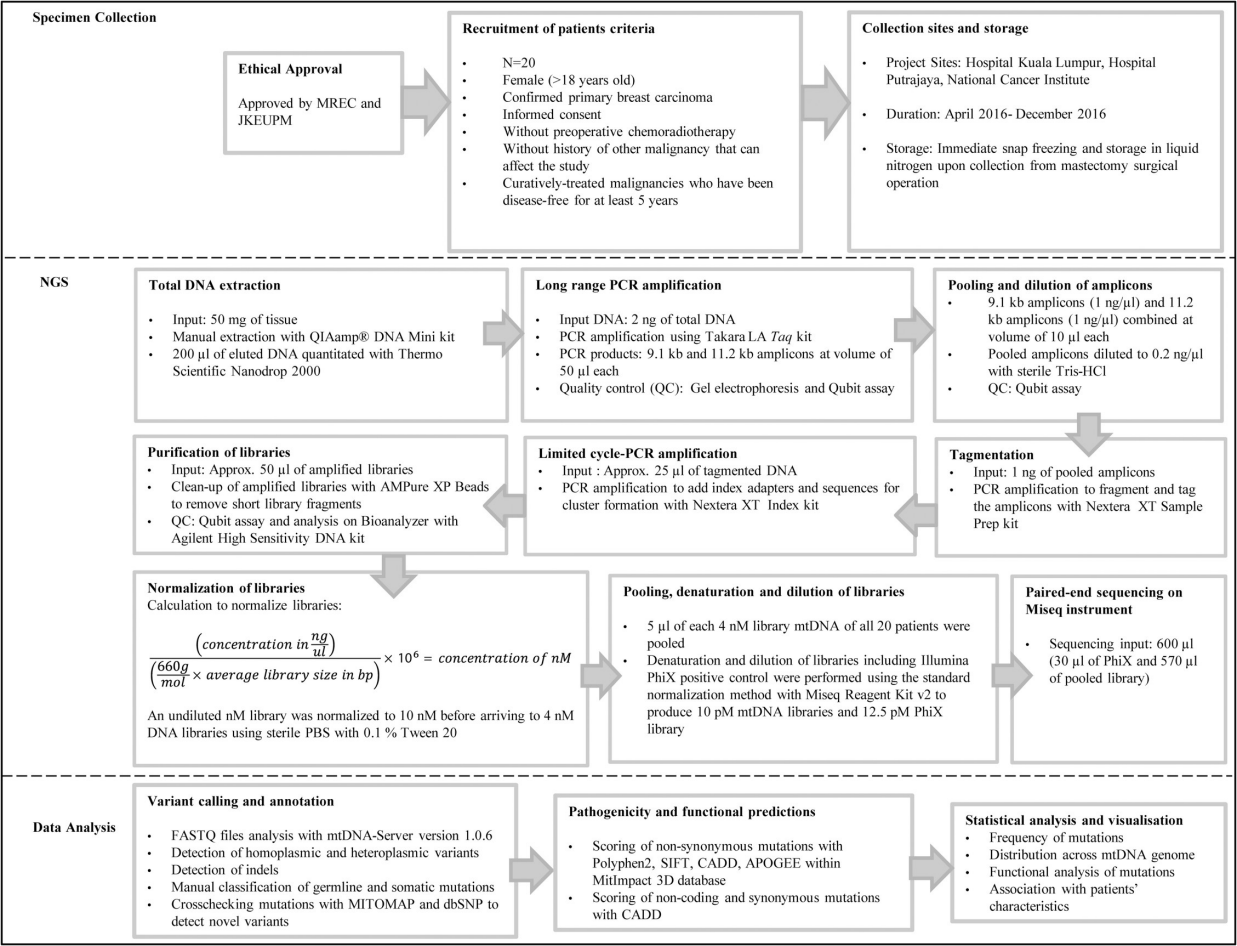
Flow chart describing the mtDNA mutation study.

The mtDNA mutation study was conducted at the Integrative Pharmacogenomics Institute (iPROMISE) in Universiti Teknologi MARA Puncak Alam Campus in Malaysia. A flow chart summarizing the methodology is shown in Fig 1.

## Results

### Patient characteristics

The corresponding normal breast tissue specimens act as controls. The mean age of patients at the time of initial surgery was 57.45 years (range 38–78 years) (S1 Table). Briefly, all tumours were invasive breast carcinomas and six of the tumours were confirmed to be triple-negative. Ten of the patients were diagnosed with stage 2, nine with stage 3 and one with stage 4 breast cancer. NGS was performed to sequence the entire mtDNA for all 20 tumours paired with normal breast tissue.

### Annotation of variants

Lists of homoplasmic and heteroplasmic variants identified by mtDNA-server for all 20 tumour-normal sample pairs are presented in Table 1 of S2 Table. Inspection of the variants led to the exclusion of mitochondrial hotspot 3017 according to the rCRS (Tables 2–5 in S2 Table). The average read depth of entire mtDNA genome for all samples was around 9500x (mean ± SEM = 9544x ± 350x). The homoplasmic and heteroplasmic variants including indels identified by the mtDNA-server were further classified as germline or somatic mutations using a variant allele fraction (VAF) threshold ≥ 1%. Variants detected in both tumour and corresponding normal tissue samples of a patient were classified as germline mutations as previously described [28, 29]. Variant differences between tumour and matched normal mtDNA were classified as somatic mutations [28, 29].

### Germline mtDNA mutations

All patients (100%) carried germline mtDNA mutations. In total, 753 germline mtDNA mutations were identified with an average of 37.7 mutations per patient. Among the germline mutations identified, 632 (83.9%) were homoplasmic single nucleotide variants (S3 Table), 63 (8.4%) were heteroplasmic single nucleotide variants (S4 Table) and 58 (7.7%) were insertions and deletions (indels) (S5 Table).

Of the homoplasmic mutations, 630 (99.7%) were known variants reported in MITOMAP [30] database, whereas two mutations (0.32%) were novel. Patients were found sharing similar homoplasmic mutations in their germline: variants A73G, A263G, A750G, A1438G, A2706G, A4769G, C7028T, A8860G, G11719A, C14766T and A15326G were detected in all patients (100%); variant T16519C was found in 70% of the population; variants C12705T and C16223T was found in 50% of the population; variants T489C, A8701G, T9540C, C10400T, T10873C, T14783C, G15043A and G15301A were found in 40% of the population; variant T16362C was found in 30% of the population; variants A827G, T6392C and G13928C were found in 20% of the population; variants G499A, C3970T, G4820A, G10310A, G12372A, G13590A, C15535T, T16136C, A16183C, T16189C, T16189C, T16217C, T16249C, C16292T and T16304C accounts for 15% of the population. The rest of the germline variant accounts for 5–10% of patient population. Of note, the current study observed A10398G germline mutation in 45% of tested population. Prevalence of A10398G polymorphism was previously reported in invasive breast cancer Malay patients in peninsular Malaysia [20]. However, the observed population consisted of Malay (30%), Chinese (20%) and Indian (5%) (S1 Fig).

**Fig 2:**
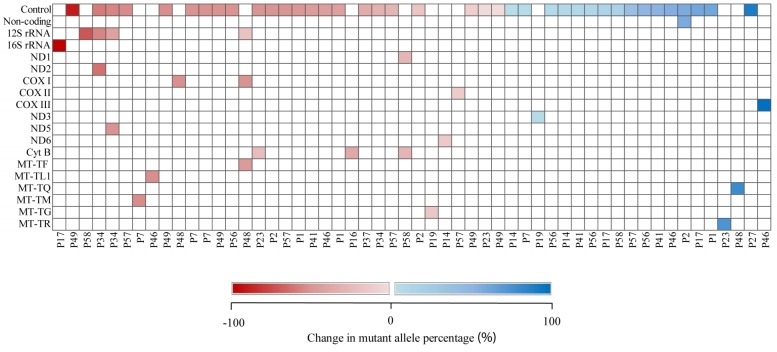
Shifts in the variant allele percentage of heteroplasmic germline variants. Each coloured rectangle corresponds to a heteroplasmic germline mutation (white = no heteroplasmy present, red = decreases in mutant allele percentage from normal to tumour, blue = increases in mutant allele percentage from normal to tumour). The shifts in heteroplasmy are presented in tabular format in S4 Table. Adapted from [34].

A total of 63 (8.4%) germline heteroplasmies at 46 distinct nucleotide positions were detected in 18 individual breast cancer patients (S4 Table). Seven of the heteroplasmies were present in multiple individuals with A189G in 44% of the population, T16189C in 22% of the population, T204C and T16093C in 16.7% of the population, G207A, G16129A and A16183C in 11.1% of the population. Of the heteroplasmic mutations, 61 (96.8%) were known variants reported in MITOMAP [30] and two others (3.2%) were novel. A mixture of reference and non-reference alleles in the same base position is regarded as heteroplasmies. The abundance of non-reference alleles ranged between 1.02% and 98.96% (median 2.72%) in normal tissue samples, whereas non-reference alleles ranged between 0.03% and 99.7% (median 1.26%) in corresponding tumour tissue samples. In overall, there was a decreasing trend towards loss of non-reference alleles or mutant alleles in the corresponding tumour samples (P = 0.13, t = 1.53) (Fig 2).

Indels were found in all patients (100%) accounting for a total of 58 indels at 7 distinct nucleotide positions (S5 Table). All germline indels have occurred in non-coding regions and have been previously reported. The majority of indels were heteroplasmic (89.7%) found at nucleotide regions of 309, 315, 514–517 and 8271–8279 base pairs. The remaining (10.3%) were homoplasmic deletions occurred in nucleotide region 249, 514–515 and 8270–8278 base pairs. Percentage of population carrying the indels is 315 ins C (95%), 309 ins C (80%), 309 ins CC (30%), 514–515 CA (35%), 249 del A (20%), 8271–8279 del (20%), 514–517 del (5%) and 8270–8278 del (5%).

### Somatic mtDNA mutations

**Fig 3:**
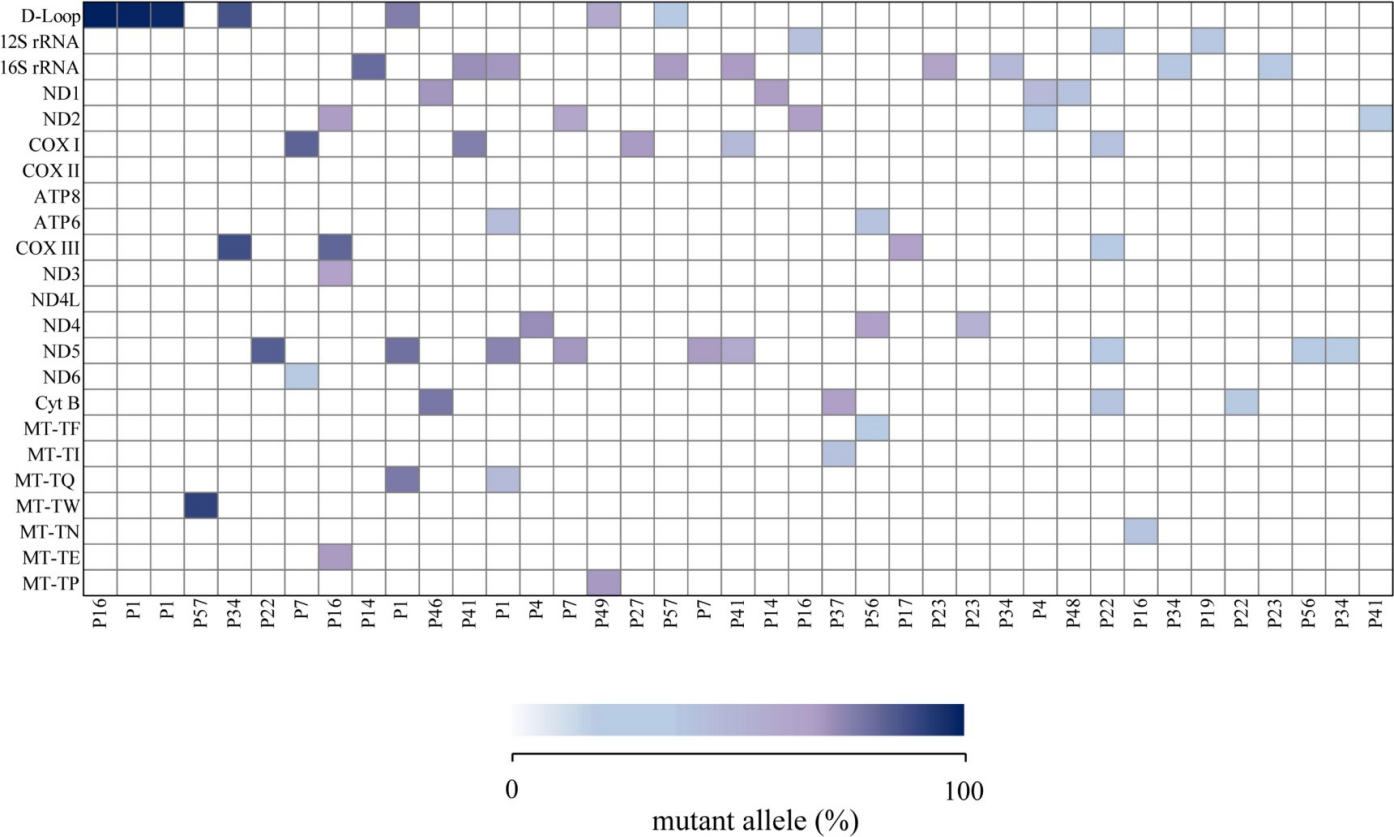
Somatic mtDNA mutations. Each coloured rectangle corresponds to a somatic mtDNA mutation, with a shading indicating mutant allele percentage (white = 0%, dark blue = 100%). Adapted from [34].

Somatic mutations were detected in 18 of 20 patients (90%). A total of 65 somatic mutations were detected, with a mean of 3.25 mutations per tumour sample. Of the 65 somatic mutations, 30 (46.2%) were novel and 35 (53.8%) were known variants in MITOMAP and dbSNP databases (Table 1). All somatic mutations were heteroplasmic in the tumour samples, 44 (67.7%) of which were low-level heteroplasmies (<10%) while 21 (32.3%) were high-level heteroplasmies (≥10%). Mutant allele abundance in the tumour samples ranged between 1.01% and 85.7% (median = 3.9%) (Fig 3). Each somatic mutation occurred only once, unique to each individual patient, and 13 (20%) of the mutations were associated with disease in previous studies (S6 Table).

**Table 1:** Somatic mtDNA mutations in breast cancer.

Patient ID	Gene	rCRS	Mutation	N > T	Variants %	AAC	Pathogenicity predictions			
							CADD	Polyphen2	SIFT	APOGEE
**P001**	D-loop	C	C150T	Homo > Hetero	80.51		Neutral			
	D-loop	A	A214G	Homo > Hetero	83.32		Neutral			
	16S rRNA	T	T2171C^a^	Homo > Hetero	10.25		Neutral			
	TI	G	G4309A	Homo > Hetero	2.24		Neutral			
	TQ	A	A4343G	Homo > Hetero	28.95		Neutral			
	ATP6	T	T8547C	Homo > Hetero	2.07	L7P	Deleterious	Probably damaging	Neutral	Neutral
	ND5	G	G12979A^a^	Homo > Hetero	34.34	G215S	Deleterious	Benign	Neutral	Neutral
	ND5	G	G13138A^a^	Homo > Hetero	22.19	E268K	Deleterious	Probably damaging	Neutral	Neutral
	D-loop	A	A16233G	Homo > Hetero	26.18		Neutral			
**P004**	ND1	C	C3738T	Homo > Hetero	2.26	V144V	Neutral			
	ND2	A	A4767G	Homo > Hetero	1.35	M100V	Neutral	Benign	Neutral	Neutral
	ND4	T	T10908C	Homo > Hetero	15.6	F50S	Neutral	Benign	Neutral	Neutral
**P007**	ND2	G	G4665A^a^	Homo > Hetero	3.6	A66T	Deleterious	Probably damaging	Neutral	Neutral
	CO1	G	G6517A^a^	Homo > Hetero	43.05	G205D	Deleterious	Probably damaging	Deleterious	Pathogenic
	ND5	T	T12365C^a^	Homo > Hetero	10.09	L10P	Deleterious	unknown	Neutral	Neutral
	ND5	T	T14102C^a^	Homo > Hetero	7.01	L589P	Deleterious	Probably damaging	Neutral	Neutral
	ND6	G	G14453A	Homo > Hetero	1.23	A74V	Deleterious	Probably damaging	Neutral	Pathogenic
**P014**	16S rRNA	G	G2701A	Homo > Hetero	37.97		Neutral			
	ND1	G	G3380A	Homo > Hetero	5.63	R25Q	Deleterious	Probably damaging	Neutral	Pathogenic
**P016**	D-loop	C	C186T	Homo > Hetero	85.65		Neutral			
	12S rRNA	G	G951A	Homo > Hetero	1.86		Neutral			
	ND2	A	A4870T^a^	Homo > Hetero	6.28	Q134L	Deleterious	Probably damaging	Neutral	Neutral
	ND2	T	T4911C	Homo > Hetero	5.15	S148P	Deleterious	Benign	Neutral	Neutral
	TN	A	A5711G	Homo > Hetero	1.62		Neutral			
	CO3	T	T9280C^a^	Homo > Hetero	41.59	L25P	Deleterious	Probably damaging	Neutral	Neutral
	ND3	T	T10076C	Homo > Hetero	3.88	I6I	Neutral			
	TE	G	G14698A^a^	Homo > Hetero	7.19		Neutral			
**P017**	CO3	G	G9838A^a^	Homo > Hetero	3.88	G211D	Deleterious	Probably damaging	Neutral	Pathogenic
**P019**	12S rRNA	A	A1528G^a^	Hetero > Hetero	1.29		Neutral			
**P022**	12S rRNA	G	G945A^a^	Homo > Hetero	1.46		Neutral			
	CO1	G	G6810A^a^	Homo > Hetero	1.68	A303T	Deleterious	Probably damaging	Neutral	Neutral
	CO3	G	G9820A	Homo > Hetero	1.08	G205E	Deleterious	Probably damaging	Neutral	Pathogenic
	ND5	A	A12612G	Homo > Hetero	45.94	V92V	Neutral			
	ND5	A	A13183G	Homo > Hetero	1.16	I283V	Deleterious	Benign	Neutral	Neutral
	CYB	G	G14869A	Homo > Hetero	1.25	L41L	Neutral			
	CYB	G	G15699A^a^	Homo > Hetero	1.6	R318H	Deleterious	Probably damaging	Neutral	Neutral
**P023**	16S rRNA	T	T2233C^a^	Homo > Hetero	3.85		Neutral			
	16S rRNA	G	G2732A^a^	Homo > Hetero	1.14		Neutral			
	ND4	G	G11226A^a^	Homo > Hetero	2.86	G156D	Deleterious	Probably damaging	Deleterious	Pathogenic
**P027**	CO1	G	G5991A	Homo > Hetero	7.72	G30S	Deleterious	Probably damaging	Deleterious	Pathogenic
**P034**	16S rRNA	A	A2623G	Homo > Hetero	2.38		Neutral			
	16S rRNA	G	G2702A	Homo > Hetero	1.35		neutral			
	CO3	G	G9868A	Homo > Hetero	56.27	R221H	Deleterious	Possibly damaging	Neutral	Pathogenic
	ND5	C	C12906T	Homo > Hetero	1.08	I190I	Neutral			
	D-loop	A	A16165G	Hetero > Hetero	53.49		Neutral			
**P037**	TI	G	G4282A	Homo > Hetero	1.68		Neutral			
	CYB	C	C15702T^a^	Homo > Hetero	4.7	P319L	Deleterious	Probably damaging	Neutral	Neutral
**P041**	16S rRNA	G	G2333A^a^	Homo > Hetero	6.7		Neutral			
	16S rRNA	G	G2815A	Homo > Hetero	15.3		Neutral			
	ND2	T	T5130C^a^	Homo > Hetero	1.04	L221L	Neutral			
	ND2	T	T5200C^a^	Homo > Hetero	2.31	I244T	Deleterious	Benign	Neutral	Neutral
	CO1	G	G6028A^a^	Homo > Hetero	25.54	G42D	Deleterious	Probably damaging	Deleterious	Neutral
	ND5	G	G13099C^a^	Homo > Hetero	3.3	A255P	Deleterious	Probably damaging	Neutral	Pathogenic
**P046**	ND1	G	G4244A^a^	Homo > Hetero	10.81	S313N	Neutral	Benign	Neutral	Neutral
	CYB	G	G14963A	Homo > Hetero	30.63	V73M	Deleterious	Probably damaging	Neutral	Neutral
**P048**	ND1	T	T4007C^a^	Homo > Hetero	1.71	M234T	Deleterious	Probably damaging	Neutral	Pathogenic
**P049**	TP	T	T15971C^a^	Homo > Hetero	8.83		Neutral			
	D-loop	C	C16295T^a^	Homo > Hetero	3.46		Neutral			
**P056**	TF	G	G627A^a^	Homo > Hetero	1.01		Neutral			
	ATP6	G	G8854A	Homo > Hetero	1.68	A110T	Deleterious	Benign	Deleterious	Neutral
	ND4	G	G10775A	Homo > Hetero	4.59	V6I	Neutral	Benign	Neutral	Neutral
	ND5	G	G12835A^a^	Homo > Hetero	1.08	A167T	Deleterious	Probably damaging	Neutral	Neutral
**P057**	D-loop	G	G513A	Homo > Hetero	1.06		Neutral			
	16S rRNA	G	G3022A	Homo > Hetero	7.58		Neutral			
	TW	G	G5521A	Homo > Hetero	37.04		Neutral			

^a^ Novel variants; N>T, normal to tumour heteroplasmic pattern; AAC, amino acid change.

### Distribution of germline and somatic mtDNA mutations across mtDNA genome

**Fig 4:**
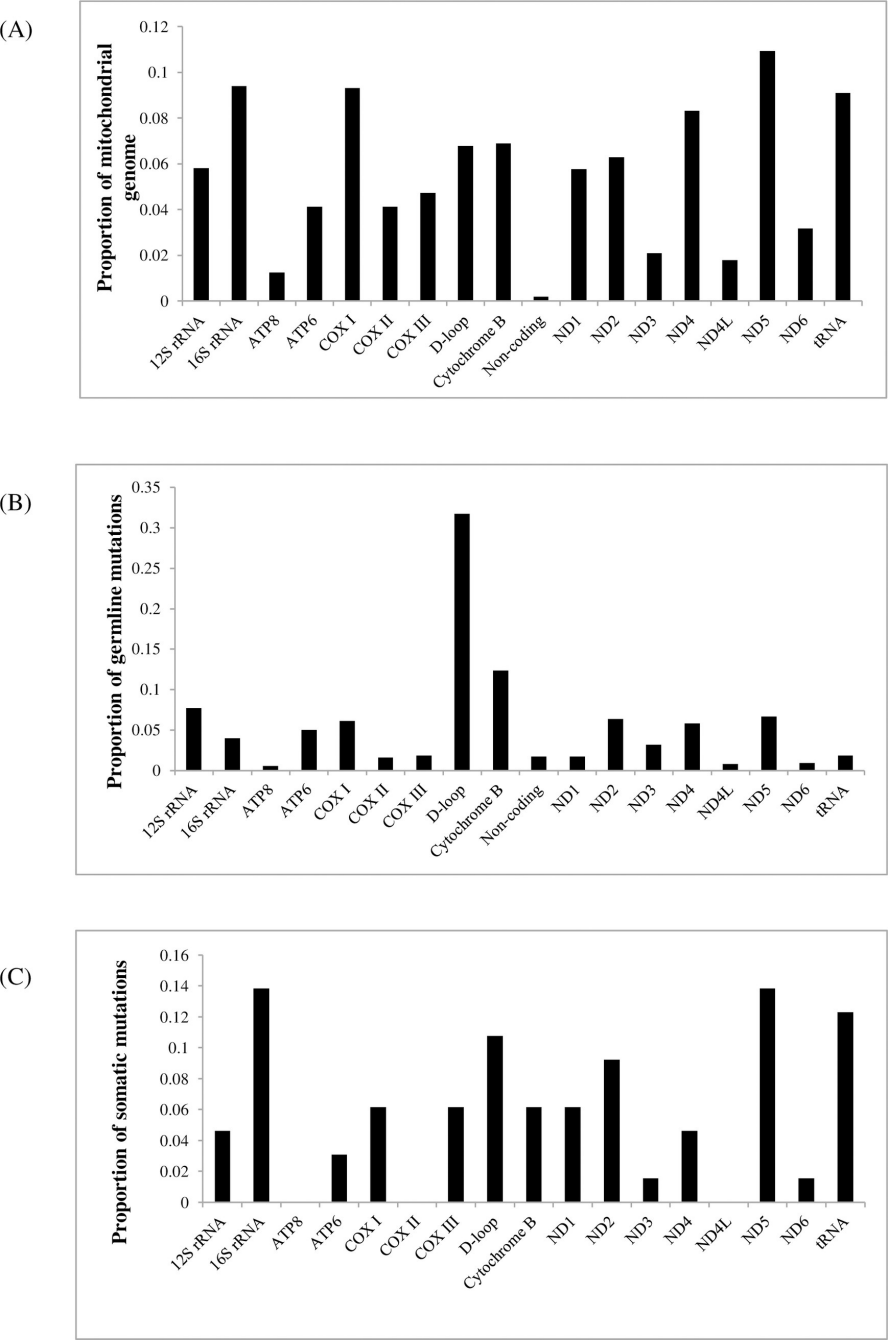
Distribution of mutations across the mtDNA genome in breast cancer patients. (A) The proportion of the 16.6kb mtDNA genome. (B) The distribution of germline mutations (Total = 753) across mtDNA genome. (C) The distribution of somatic mutations (Total = 65) across mtDNA genome.

Overall, 245 (32.5%) of germline mutations were within the D-loop region, 399 (53.0%) were within protein-coding region, 88 (11.7%) were within rRNA region, 14 (1.9%) were within tRNA region and 7 (0.9%) were within other non-coding regions. A similar pattern of distribution was observed for somatic mutations whereby 7 (10.8%) of somatic mutations were within the D-loop region, 38 (58.5%) were within protein-coding region, 12 (18.5%) were within rRNA region and 8 (12.3%) were within tRNA region. The proportional distribution of germline and somatic mtDNA mutations across the mtDNA genome are depicted in Fig 4. The differences in distribution of mutations across the mtDNA genome between germline and somatic mutations is highly significant (Fisher’s P<0.001). Additionally, among the protein-coding genes, the proportional number of somatic mutations correlated strongly with the proportional transcript size of the gene in relation to the entire mitochondrial genome (Pearson r = 0.84; P = 0.01). This suggests that somatic mutations in the protein coding genes occur in respect to the length of gene in the mtDNA genome of breast cancer patients. When the number of mutations was normalized to the size of the gene region, it was found that the D-loop region showed 2× and 6× susceptibility to somatic and germline events in breast cancer, respectively.

Given the role of protein-coding genes in the OXPHOS for the generation of cellular energy, the distribution of somatic and germline mutations within the OXPHOS cascade protein complexes was studied. Table 2 shows the distribution of somatic and germline mutations among OXPHOS components (complexes I, III, IV and V) encoded by mtDNA. There are no major variations in mutation distribution between the germline and somatic mutations (Fisher P = 0.159). The Complex I genes, however have the largest number of somatic mutations (36.9%) and germline mutations (25.5%). Interestingly, ND5 gene of Complex I harboured the largest number of somatic (37.5%) and germline mutations (26.0%) (Fig 4), consistent with a previous breast cancer study [34]. Complex III had (6.2%) somatic mutations and (12.4%) germline mutations, while Complex IV had (12.3%) somatic and (9.6%) germline mutations. The least number of mutations was observed in complex V genes, with (3.1%) of somatic mutations and (5.6%) of germline mutations.

**Table 2:** Distribution of somatic and germline mtDNA mutations in the OXPHOS components.

Region	Germline Mutation N (%)	Somatic Mutation N (%)
**Complex I**	192 (25.5)	24 (36.9)
**Complex III**	93 (12.4)	4 (6.2)
**Complex IV**	72 (9.6)	8 (12.3)
**Complex V**	42 (5.6)	2 (3.1)

### Impact of mtDNA mutations

**Fig 5:**
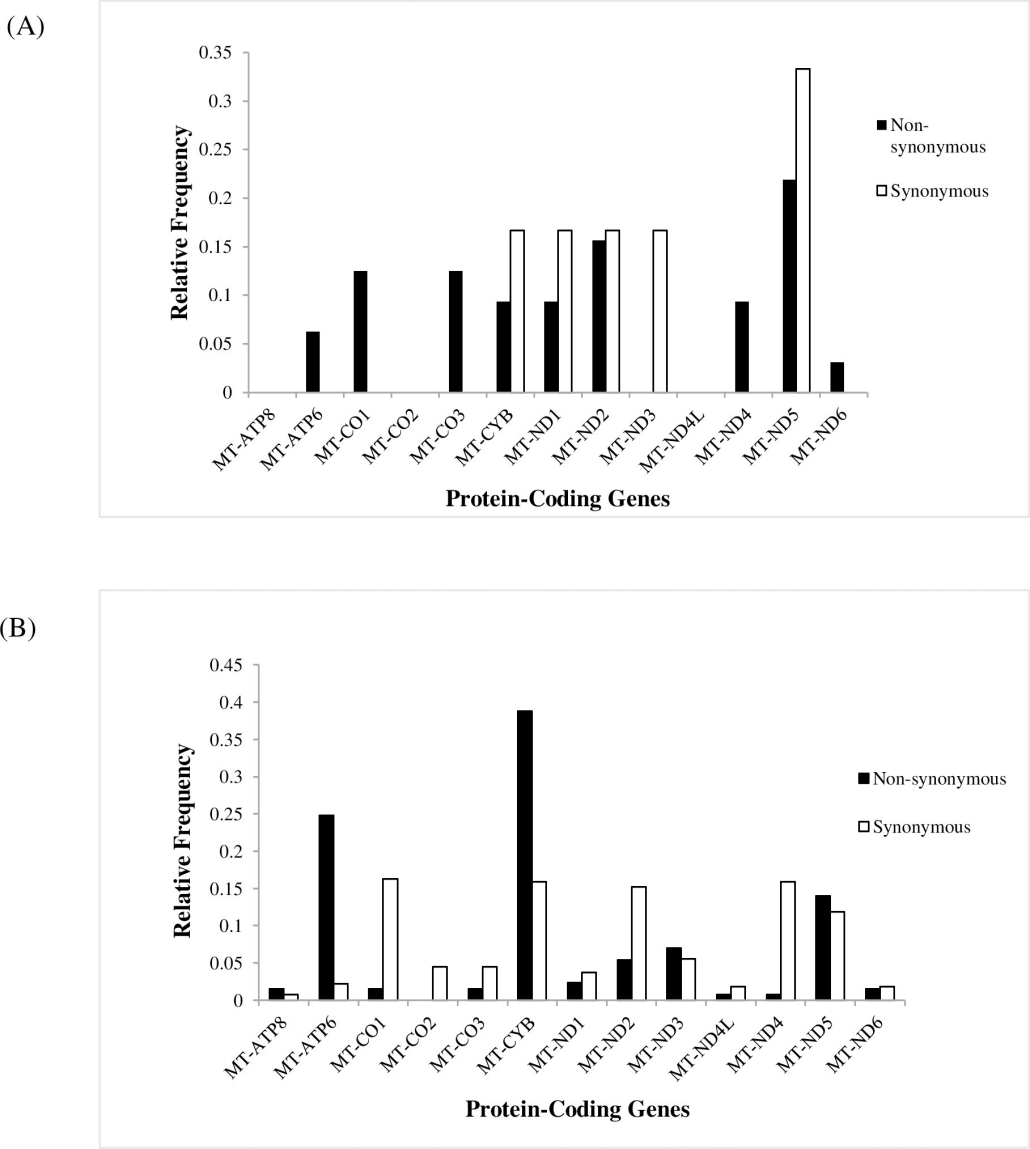
Relative frequencies of non-synonymous and synonymous mutations across mtDNA protein-coding genes. (A) Somatic mtDNA mutations; (B) Germline mtDNA mutations.

Of the 38 somatic mutations observed in protein-coding regions, 32 (84.2%) were non-synonymous while 6 (15.8%) were synonymous (Fig 5A). In contrast, of the 399 germline mutations observed in the protein-coding regions, 129 (32.3%) were non-synonymous while 270 (67.7%) were synonymous (Fig 5B). There was a significant enrichment of non-synonymous somatic mutations as compared with germline mutations (Fisher P<0.0001). Based on the overall deleterious and functional prediction scores through CADD, Polyphen2, SIFT and APOGEE, 10 missense somatic mutations were potentially damaging (see Table 1). Mutations occurred predominantly in Complex I (15.6%) and Complex IV (15.6%) genes. Comparatively, there are only five homoplasmic germline mutations known to cause deleterious protein function (S3 Table). The frequency of these mutations within the tested population was M1T (5%), L237M (10%), L17F (10%), F26S (5%) and N333I (5%). Mutations L237M and L17F were found to occur in two cases, P017 and P058. Interestingly, patients P017 and P058 were found to share the major haplogroup D. Based on Mitomap, the two mutations have been previously associated to other diseases but not with cancer. In addition, less severe missense mutations T333A and I78T, as well as neutral mutations P183P, P36P and M2M, were found in the same two patients with major haplogroup D. Further study in a larger population is required to understand the significance of haplogroup D mutations as potential biomarkers in breast cancer. The identity of the mtDNA haplogroup of all patients can be found listed in Table 1 of S2 Table. A venn diagram of tested population with the major haplogroups is listed in S2 Fig.

In finding the possible role of tRNA mutations found in the current study, somatic mutations G4309A, G4282A and G5521A were classified as pathogenic when assessed with MitoTIP [35]. Each of these mutations occurred in 5% of the tested population. Eight (1.3%) germline homoplasmic tRNA mutations and 6 (22.2%) germline heteroplasmic tRNA mutations were mostly identified as benign mutations (92.9%) in the MitoTIP [35]. Each of these germline tRNA mutations was found to occur in 5% of the population studied.

The functional impact of mutations in the rRNA genes is currently unknown. However, differences in the occurrence of somatic and germline mutations in rRNA genes in the current study is worthy of future investigation. It was found that among 12 (18.5%) somatic mutations in the rRNA genes, a predominant (13.8%) of mutations occurred in the 16S rRNA gene region. Whereas, among 88 (12.7%) germline mutations identified in the rRNA genes (65.9%) were predominantly found in the 12S rRNA gene region.

As reported earlier, the D-loop contains a substantial number of somatic (10.8%) and germline mutations (31.9%) in comparison to other genes despite its small size and non-coding properties. The somatic D-loop mutations in 25% of the population, most commonly occurred in the hypervariable region 1 (HVR-1) (4.6%) and HVR-2 (4.6%). Similarly, germline D-loop mutations in all patients (100%) occurred largely in the HVR-1 (54.7%) and HVR-2 (37.0%). Somatic mutations in the HVR-2 especially C150T, C186T and A214G as well as germline mutations from nucleotide positions 146–374 are likely to affect heavy-strand synthesis as they were located at the origin of heavy-strand replication (OHR) sites.

### Association with clinicopathological parameters

The association between the frequency of somatic and germline mtDNA mutations with patients’ age, ethnicity, tumour grades and stages, nodal stages and hormone receptors in breast cancer were investigated. Significant association was found between frequency of somatic mutations in the rRNA gene region and tumour grades (P = 0.01) and tumour stages (P = 0.022). Breast tumour grade III (73%) appeared to express more rRNA somatic mutation than the grade II tumours (11%), while stage III breast tumours (78%) carried more rRNA somatic mutation than the other tumour stages. In addition, the frequency of somatic mutations in the protein coding genes was significantly different between different nodal stages in breast cancer (P = 0.016) compared to other gene regions. An overall lack of associations with various other clinicopathological variables may be attributed to the limited sample size for the current study. S7 Table describes the mtDNA mutation status and its association with patients’ characteristics.

## Discussion

MtDNA mutations occur frequently in cancer and have recently emerged as non-invasive cancer biomarkers for evaluating the risk and prognosis of the disease [36, 37]. Various mutations in the coding and non-coding regions of mtDNA are associated with an increased risk of breast cancer [38]. To date, the current study is the first evidence of germline and somatic mtDNA genome mutations in Malaysian breast cancer patients.

The D-loop region is the most studied mtDNA variants as it possesses high mutation rate, associated to later stages of cancer and poor prognosis in breast cancer [39]. Several somatic mtDNA mutations in breast cancer were reported to be accumulated in the hypervariable regions HVR1 and HVR2 within the D-loop region [28]. All indels in this study are germline mutations localized in the non-coding regions. D-loop showed higher susceptibility to both germline (6×) and somatic mutations (2×) compared to other regions. However, no associations were derived in relation to stages of cancer. The CADD scores in this study showed none of the D-loop mutations were found to be deleterious and it was noted both germline and somatic mutations in the D-loop occur preferentially in HVR-1 and HVR-2. This is consistent with a previous study that supports hypervariable sites in the mtDNA control region as mutational hotspots [40].

Analysis of mutations in the coding region showed that genes encoding for OXPHOS components that regulate energy production in cells were mostly affected. Complex I genes carried the highest number of mutations as described in Table 2. The substantial amount of somatic mutations in Complex I were previously reported with ND5 subunit among Complex I genes harbouring the most somatic mutations [31, 34]. Similarly, the current study observed highest frequencies of somatic mutations (37.5%) in the ND5 subunit alone. Among the ND5 somatic mutations, 29.2% were missense deleterious mutations (Table 1). Alterations in the ND5 subunit were postulated to disrupt the Complex I assembly leading to resistance in apoptosis and ultimately promoting tumour formation and growth [41]. The current evidence suggests that breast tumours may select for mutations within functional regions of the mitochondrial genome which modulates altered metabolism and support tumorigenesis. Nonetheless, further investigation is necessary to determine the effect of ND5 somatic mutations. In this study, germline mutation A10398G was identified as a non-synonymous mutation that is benign indicated by the low pathogenicity score (S3 Table). This mutation was found in Chinese (20%) and Indian (5%) patients with breast cancer. Interestingly, A10398G in ND3 subunit has been previously reported to be a significant polymorphism (OR = 2.29, P = 0.007) linked to increased risk of invasive breast cancer in the Malay population of Peninsular Malaysia [19]. The potential use of 10398G as a biomarker in invasive breast cancer among Malaysian population warrants for further investigation.

In conclusion, the screening of mtDNA in a small cohort of patients with breast cancer in Malaysia identified germline and somatic mtDNA mutations. Evaluation for pathogenicity and functionality suggest mtDNA alterations affect protein functions. It was noted somatic mtDNA mutations were enriched for nonsynonymous changes compared to germline mutations. The findings from this study will serve as a basis in understanding mitochondrial genome of breast cancer in Malaysian population.

## Supporting information

S1 Table Demographic details and clinical features of analysed breast tissue samples. (XLSX)

S2 Table All variants identified by mtDNA-Server. (XLSX)

S3 Table Germline homoplasmic mutations with pathogenicity and functional predictions. (XLSX)

S4 Table Germline heteroplasmic mutations with pathogenicity and functional predictions. (XLSX)

S5 Table Germline indels. (XLSX)

S6 Table Somatic mtDNA mutations with pathogenicity and functional predictions. (XLSX)

S7 Table Association between frequency of mutations and patients’ characteristics. (XLSX)

S1 Fig Venn diagram showing germline mutations in Malay, Chinese and Indian patients. (PDF)

S2 Fig Venn diagram showing major haplogroups in Malay, Chinese and Indian patients. (PDF)
