# Supplementary material for: Mitochondrial DNA mutations in Malaysian female breast cancer patients
Source: PLoS One. 2020 May 22;15(5):e0233461. doi: 10.1371/journal.pone.0233461 (PMC7244147; doi:10.1371/journal.pone.0233461)
Supplement: S1 Fig — (PDF) [file pone.0233461.s008.pdf]

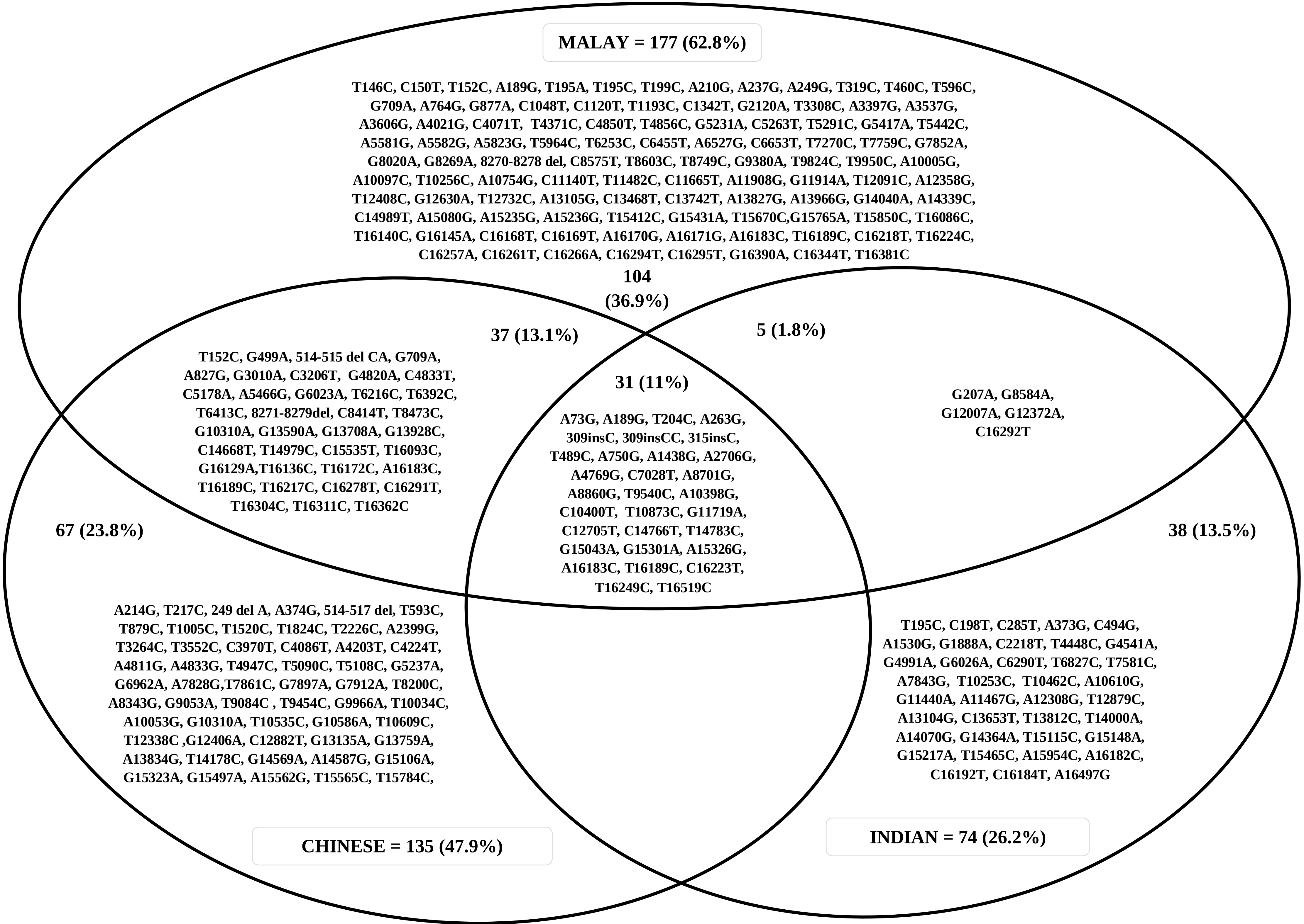
MALAY = 177 (62.8%)
T146C, C150T, T152C, A189G, T195A, T195C, T199C, A210G, A237G, A249G, T319C, T460C, T596C, G709A, A764G, G877A, C1048T, C1120T, T1193C, C1342T, G2120A, T3308C, A3397G, A3537G, A3606G, A4021G, C4071T, T4371C, C4850T, T4856C, G5231A, C5263T, T5291C, G5417A, T5442C, A5581G, A5582G, A5823G, T5964C, T6253C, C6455T, A6527G, C6653T, T7270C, T7759C, G7852A, G8020A, G8269A, 8270-8278 del, C8575T, T8603C, T8749C, G9380A, T9824C, T9950C, A10005G, A10097C, T10256C, A10754G, C11140T, T11482C, C11665T, A11908G, G11914A, T12091C, A12358G, T12408C, G12630A, T12732C, A13105G, C13468T, C13742T, A13827G, A13966G, G14040A, A14339C, C14989T, A15080G, A15235G, A15236G, T15412C, G15431A, T15670C,G15765A, T15850C, T16086C, T16140C, G16145A, C16168T, C16169T, A16170G, A16171G, A16183C, T16189C, C16218T, T16224C, C16257A, C16261T, C16266A, C16294T, C16295T, G16390A, C16344T, T16381C
104
(36.9%)
37 (13.1%)
T152C, G499A, 514-515 del CA, G709A, A827G, G3010A, C3206T, G4820A, C4833T, C5178A, A5466G, G6023A, T6216C, T6392C, T6413C, 8271-8279del, C8414T, T8473C, G10310A, G13590A, G13708A, G13928C, C14668T, T14979C, C15535T, T16093C, G16129A,T16136C, T16172C, A16183C, T16189C, T16217C, C16278T, C16291T, T16304C, T16311C, T16362C
5 (1.8%)
G207A, G8584A, G12007A, G12372A, C16292T
31 (11%)
A73G, A189G, T204C, A263G, 309insC, 309insCC, 315insC, T489C, A750G, A1438G, A2706G, A4769G, C7028T, A8701G, A8860G, T9540C, A10398G, C10400T, T10873C, G11719A, C12705T, C14766T, T14783C, G15043A, G15301A, A15326G, A16183C, T16189C, C16223T, T16249C, T16519C
67 (23.8%)
38 (13.5%)
A214G, T217C, 249 del A, A374G, 514-517 del, T593C, T879C, T1005C, T1520C, T1824C, T2226C, A2399G, T3264C, T3552C, C3970T, C4086T, A4203T, C4224T, A4811G, A4833G, T4947C, T5090C, T5108C, G5237A, G6962A, A7828G,T7861C, G7897A, G7912A, T8200C, A8343G, G9053A, T9084C , T9454C, G9966A, T10034C, A10053G, G10310A, T10535C, G10586A, T10609C, T12338C ,G12406A, C12882T, G13135A, G13759A, A13834G, T14178C, G14569A, A14587G, G15106A, G15323A, G15497A, A15562G, T15565C, T15784C,
T195C, C198T, C285T, A373G, C494G, A1530G, G1888A, C2218T, T4448C, G4541A, G4991A, G6026A, C6290T, T6827C, T7581C, A7843G, T10253C, T10462C, A10610G, G11440A, A11467G, A12308G, T12879C, A13104G, C13653T, T13812C, T14000A, A14070G, G14364A, T15115C, G15148A, G15217A, T15465C, A15954C, A16182C, C16192T, C16184T, A16497G
CHINESE = 135 (47.9%)
INDIAN = 74 (26.2%)
