# Supplementary figures and images for: Mitochondrial DNA mutations in Malaysian female breast cancer patients
Source: PLoS One. 2020 May 22;15(5):e0233461. doi: 10.1371/journal.pone.0233461 (PMC7244147; doi:10.1371/journal.pone.0233461)

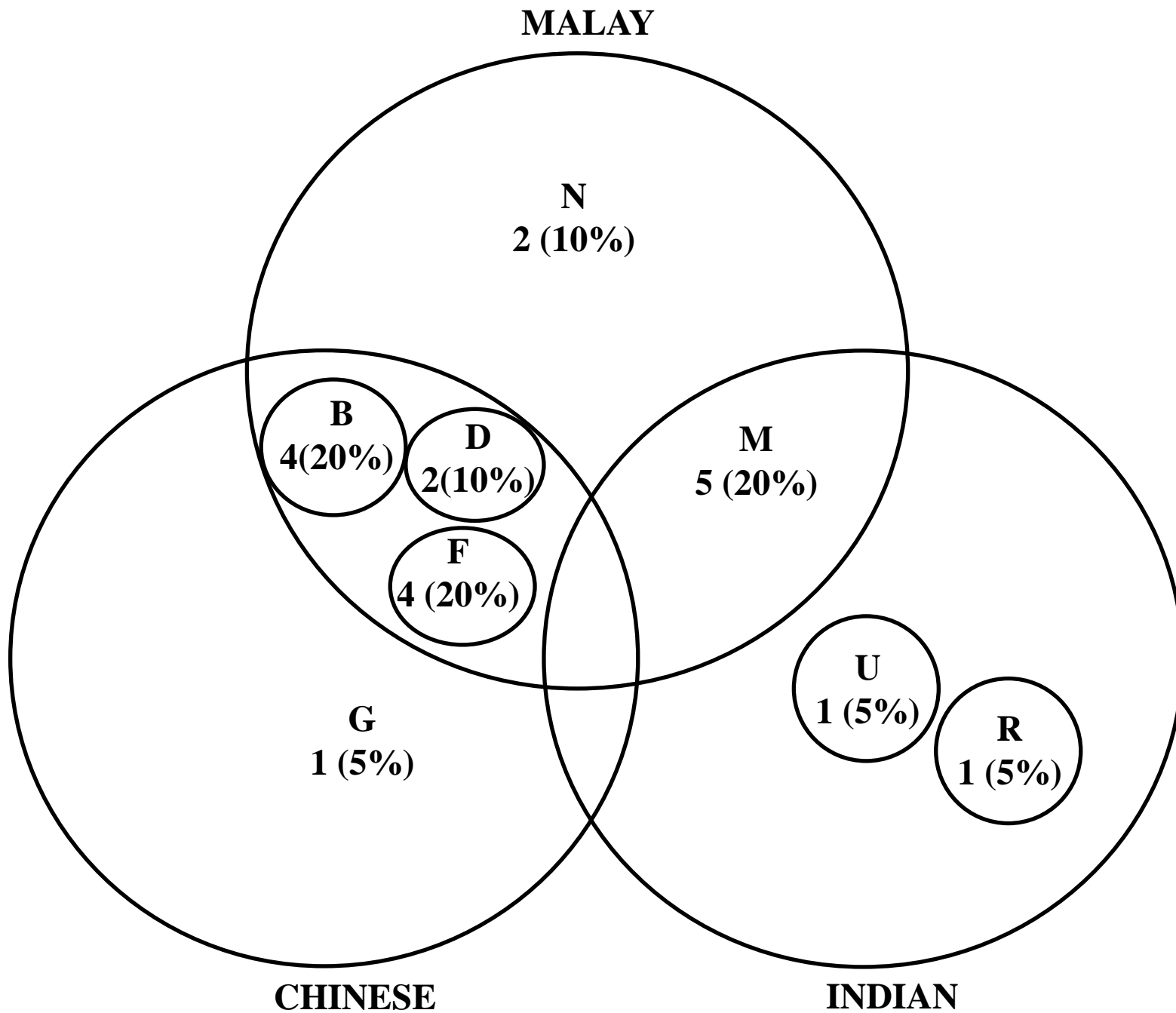

Supplement: S2 Fig — (PDF) [file pone.0233461.s009.pdf]
